# Supplementary material for: Small Airway Disease in Pulmonary Hypertension—Additional Diagnostic Value of Multiple Breath Washout and Impulse Oscillometry
Source: J Clin Med. 2018 Dec 9;7(12):532. doi: 10.3390/jcm7120532 (PMC6306708; doi:10.3390/jcm7120532)
Supplement: Supplementary file 1 [file jcm-07-00532-s001.zip › jcm-398542-SI.pdf]

Acceptability Criteria for MBW

- breathing pattern
  - stable tidal volume, no hyperventilation, no hypoventilation
  - no coughing
  - assessed clinically and in-silico
- variability
  - within 10% from the median FRC / LCI across triplicate tests
  - careful examination for technical issues if larger, but within 25%
  - rejection if larger > 25%
- no leak
  - equilibration between inspiratory and expiratory SF_6 concentrations during wash-in
  - no sudden drop in inspiratory SF_6 concentration during wash-in
- test termination
  - at least three consecutive breaths with end tidal SF_6 concentrations <1/40th of starting value
- test conduction
  - no excessive swallowing (clinical assessment)
  - sufficient interval between tests

    (twice the wash-out time or measurement of resituated gas concentrations)

Parameters for propensity score matching

- non-healthy controls (previous collective, n=367)
  - age, gender, weight, height
  - COPD, sarcoidosis, asthma, pulmonary fibrosis
  - FEV_1/VC, RV/TLC, TLCO/VA
- healthy controls (previous collective, n=34)
  - age, gender, weight, height

Table S1. Reversibility testing.

	PH (n = 25)		Non-Healthy Controls (n = 25)		
	n	%	n	%	p-value #
**test count**	12	100	9	100	0.57
			Positive		
**spirometry** §	1	8	1	11	>0.9
**body** †	2	17	3	33	0.71
**IOS** ‡	4	33	1	11	0.60

§ FEV_1: >12% and 200 ml increase according to [32], † RV: >20% decrease according to [33], ‡ AX: >50% decrease according to [34]. # Chi-squared test. * Statistically significant $p < 0.05$.

Table S2. Number of successful MBW measurements.

	PH (n = 25)		Healthy Controls (n = 25)			Non-Healthy Controls (n = 25)		
	n	%	n	%	p-Value #	n	%	p-Value #
LCI								
**0**	0	0	0	0	>0.9	0	0	>0.9
**1**	0	0	0	0		0	0	
**2**	6	24	5	20		6	24	
**3**	19	76	20	80		19	76	
S_{acin}, S_{cond}								
**0**	4	16	4	16	0.72	8	32	0.38
**1**	3	12	6	24		3	12	
**2**	10	40	9	36		5	20	
**3**	8	32	6	24		9	36	

LCI: lung clearance index, S_{acin}: acinar ventilation heterogeneity, S_{acin}: conductive ventilation heterogeneity. #Chi-squared test as compared to the PH group. * Statistically significant $p < 0.05$.

Table S3. Comorbidities (PH group, n = 25).

	n	%
arterial hypertension	17	68
atrial fibrillation	14	56
coronary heart disease	11	44
diabetes mellitus (type II)	7	28
COPD	7	28
sarcoidosis	5	20
bronchial asthma	2	8
fibrosis	2	8

COPD: chronic obstructive pulmonary disease.

A

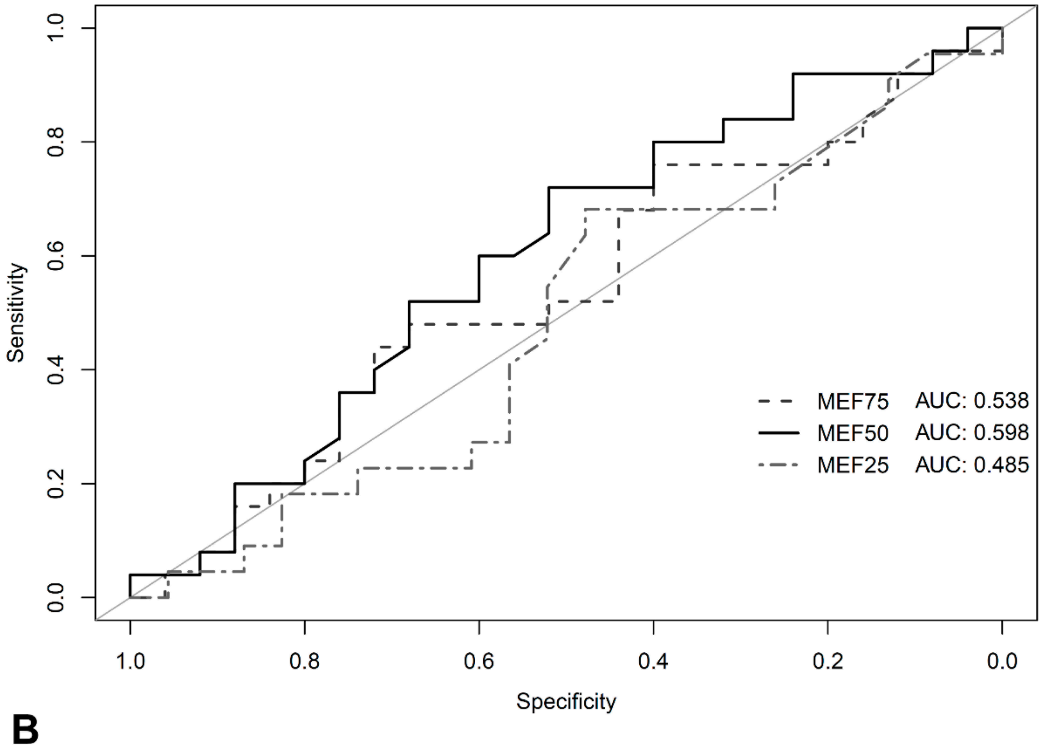

B

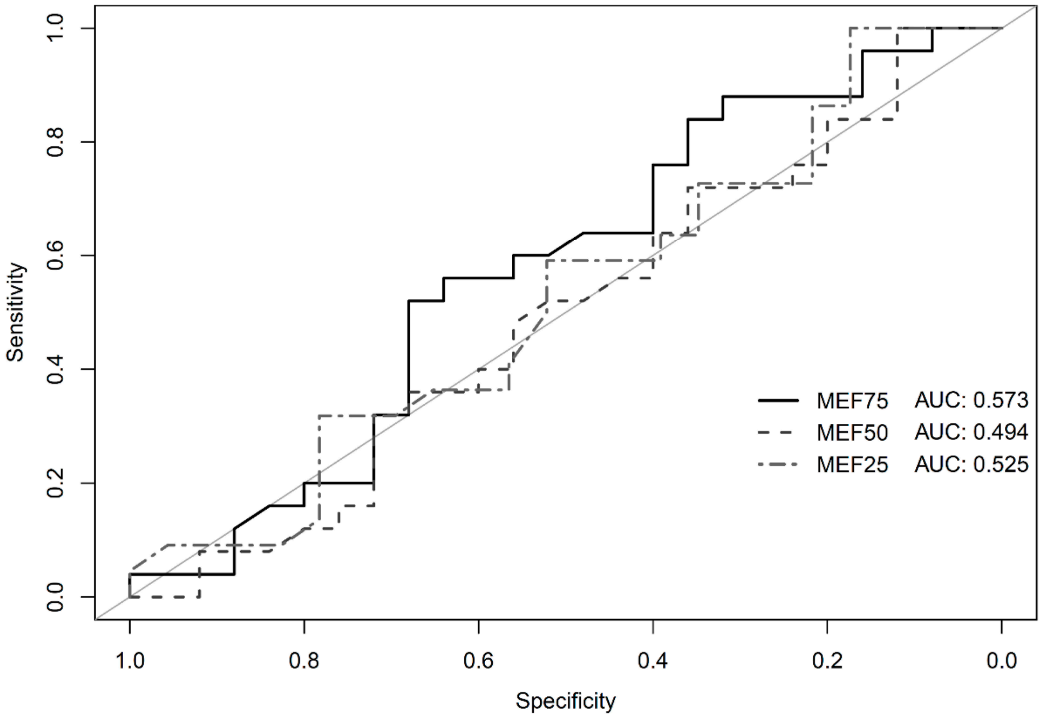

Figure S1. Diagnostic performance: ROC analysis for maximal expiratory flow (MEF) at 75, 50 and 25% of vital capacity (VC). **A**: patients with PH vs. healthy controls. **B:** patients with PH vs. non-healthy controls.
